# L-Lactate Regulates the Expression of Synaptic Plasticity and Neuroprotection Genes in Cortical Neurons: A Transcriptome Analysis

**DOI:** 10.3389/fnmol.2018.00375

**Published:** 2018-10-10

**Authors:** Michael B. Margineanu, Hanan Mahmood, Hubert Fiumelli, Pierre J. Magistretti

**Affiliations:** ^1^Laboratory for Cellular Imaging and Energetics, Biological and Environmental Sciences and Engineering Division, King Abdullah University of Science and Technology, Thuwal, Saudi Arabia; ^2^Center for Psychiatric Neuroscience, Department of Psychiatry, Lausanne University Hospital, Lausanne, Switzerland

**Keywords:** lactate, synaptic plasticity, neuroprotection, NMDA receptor, transcriptome, NADH, gene expression

## Abstract

Lactate, a product of aerobic glycolysis in astrocytes, is required for memory formation and consolidation, and has recently emerged as a signaling molecule for neurons and various cell types in peripheral tissues. In particular lactate stimulates mRNA expression of a few plasticity-related genes. Here, we describe a RNA-seq study that unravels genome-wide transcriptomic responses to this energy metabolite in cortical neurons. Our results show that mRNA expression of 20 immediate-early genes involved in the MAPK signaling pathway and in synaptic plasticity were increased by more than twofold following 1 h of lactate stimulation. This effect was dependent on NMDA receptor (NMDAR) activity since it was prevented by pre-treatment with MK-801. Comparison with published datasets showed that a significant proportion of genes modulated by lactate were similarly regulated by a stimulation protocol activating specifically synaptic NMDARs known to result in upregulation of pro-survival and downregulation of pro-death genes. Remarkably, transcriptional responses to lactate were reproduced by NADH (for 74 of the 113 genes, FDR < 0.05), suggesting a redox-dependent mechanism of action. Longer-term gene expression changes observed after 6 h of lactate treatment affected genes involved in regulating neuronal excitability and genes coding for proteins localized at synapses. Gene set enrichment analyses performed with ranked lists of expressed genes revealed effects on molecular functions involved in epigenetic modulation, and on processes relevant to sleep physiology and behavioral phenotypes such as anxiety and hyperactivity. Overall, these results strengthen the notion that lactate effectively regulates activity-dependent and synaptic genes, and highlight new signaling effects of lactate in plasticity and neuroprotection.

## Introduction

Lactate is a metabolic end-product of glycolysis with established roles as an energy substrate in multiple tissues, including skeletal muscle, heart, liver, and the brain (Adeva-Andany et al., 2014). In the brain, lactate is mainly produced by astrocytes, a type of glial cells, and shuttled to neurons in response to synaptic activity – a mechanism known as the astrocyte-to-neuron L-lactate shuttle, ANLS (Pellerin and Magistretti, 1994, 2012).

In addition to its energetic role, L-Lactate has emerged as a valuable signaling molecule in different tissues and cell types, including circulating stem/progenitor cells (SPCs) (Milovanova et al., 2008), muscle (Hashimoto et al., 2007), T cells, monocytes and dendritic cells (Gottfried et al., 2006; Dietl et al., 2010; Haas et al., 2016). Lactate is also an active metabolite for cancer cells promoting angiogenesis, immune escape and cell migration (Kim and Dang, 2006; San-Millan and Brooks, 2017).

Recent work has demonstrated that lactate also acts as a signaling molecule in the brain (Magistretti and Allaman, 2018), modifying the excitability of neurons (Tang et al., 2014; Sada et al., 2015), playing an important role in learning and memory (Suzuki et al., 2011) and favoring neuroprotection (Jourdain et al., 2016).

The essential role of lactate in long-term memory formation in mice (Suzuki et al., 2011) is likely to rely on modulation of synaptic plasticity and neuronal activity-dependent genes. Yang et al. (2014) have shown in primary cultured neurons and in the mouse sensory motor cortex that L-lactate stimulates the expression of genes such as *Arc, c-Fos, Bdnf* and *Zif268* (*Egr1*). These genes are important for synaptic plasticity and are induced by neuronal activity (Guzowski et al., 2001; Flavell and Greenberg, 2008). The effect of lactate on these genes was dependent in particular on NMDA receptor (NMDAR) activity, being abolished in the presence of NMDAR antagonist MK801.

NMDA receptors are glutamate-gated ion channels with important roles in excitatory neurotransmission, experience-dependent synaptic remodeling, and long-lasting changes in synaptic efficacy such as LTP, a key process in learning and memory (Cull-Candy and Leszkiewicz, 2004; Lynch, 2004; Lau and Zukin, 2007).

Lactate was also shown to regulate gene expression in other cell systems and tissues, such as L6 muscle cells (Hashimoto et al., 2007), HCT 116 colon cancer cells (Latham et al., 2012), glioma cells (Baumann et al., 2009), tumor-associated macrophages (Colegio et al., 2014), T cells (Haas et al., 2015), and mesenchymal stem cells (Zieker et al., 2008).

In the context of the observed effects and in order to gain further insights into the signaling mechanisms regulated by lactate in the brain, we have evaluated genome-wide transcriptional responses to this metabolite in primary neuron cultures.

Results show that L-lactate modulates key genes important for different forms of synaptic plasticity in an NMDAR-dependent manner and genes under the genomic pro-survival program activated specifically by synaptic NMDARs. Furthermore, NADH, but not pyruvate, primarily reproduces these effects on gene expression. In addition, functional bioinformatics analysis of gene expression data points to the involvement of the MAPK signaling pathway, the serum response factor (SRF) and transcription factor Zif268 (Egr1).

The transcriptome study also unravels groups of genes modulated by L-lactate in an NMDAR-independent manner, for which other signaling pathways and gene regulatory mechanisms could be responsible. These results open avenues for further investigations that can contribute to a better understanding of the physiological coupling of energy metabolism to synaptic activity in the brain.

## Materials and Methods

### Reagents

Sodium L-lactate and sodium L-pyruvate were purchased from Sigma-Aldrich. (5S,10R)-(+)-5-methyl-10,11-dihydro-5*H*-dibenzo[a,d]cyclohepten-5,10-imine maleate (MK801 maleate) and *N*-methyl-D-aspartic acid (NMDA) were purchased from Tocris. NADH was obtained from Roche.

### Cell Cultures

Experiments were conducted in accordance with the Swiss Federal Guidelines for Animal Experimentation and were approved by the Cantonal Veterinary Office for Animal Experimentation (Vaud, Switzerland). Primary cultures of cortical neurons were prepared from embryonic day 17 (E17) OF1 mice embryos (Charles River Laboratories) as previously described (Allaman et al., 2010). Briefly, minced pieces (1–2 mm^3^) of isolated cerebral cortices were enzymatically digested with papain and gently triturated to a single-cell suspension. Dissociated cells were plated onto poly-L-ornithine-coated cell culture dishes at a density of 4 × 10^4^ cells/cm^2^ and maintained in Neurobasal medium supplemented with B27, GlutaMax, penicillin (50 U/mL), and streptomycin (50 μg/mL) (Invitrogen) at 37°C in a humidified atmosphere of 5% CO_2_ and 95% air. These culture conditions typically produced 93–95% pure neuronal cultures (Belanger et al., 2011). Neurons were used for experiments at day *in vitro* 11.

### Cell Treatments

Neuronal cultures were treated by directly adding lactate (20 mM), pyruvate (20 mM), NADH (4 mM) or NMDA (50 μM) into the culture medium from 50 to 100x stock solutions prepared in milli-Q^®^ water. When indicated, MK-801 (40 μM) was added 30 min prior to lactate. For each independent culture and treatment condition, three biological replicates were included.

### RNA Extraction and Sequencing

Total RNA was isolated from cultured cells using Nucleospin RNA II kit (Macherey-Nagel) according to the manufacturer’s instructions. As much as 5 μg of RNA, quantified using NanoDrop spectrophotometer (Nanodrop 2000, Thermo Scientific), were preserved in RNAstable (Biomatrica) and subsequently shipped to KAUST’s next-generation sequencing core lab where it was reconstituted in RNAse-free water. The integrity and level of degradation of the purified RNA was assessed with the Agilent BioAnalyzer 2100 (Agilent Technologies, Inc.). RIN value was above 8 for all the samples, on a scale from 1 (degraded) to 10 (intact).

Illumina RNA-Seq was performed according to manufacturer’s instructions. Library was prepared using the TruSeq stranded mRNA library protocol. Briefly, mRNA was isolated from total RNA with poly-T oligo-attached magnetic beads and then fragmented. First-strand cDNA was synthesized using SuperScript III reverse transcriptase and random primers. Second strand cDNA was subsequently synthesized and double-stranded cDNA generated. The cDNA was adenylated and ligated to adapters. cDNA fragments were purified and size-selected with AMPure XP beads (Beckman Coulter).

Sequencing was performed on the HiSeq^TM^ 2000 (Illumina) with a paired-end (PE) sequencing strategy. The read length was set at 100 bp (PE) and the sequencing depth was 40–50 million reads.

### qRT-PCR

The first strand of cDNA was synthesized from 1,000 ng of total RNA (10 min at 25°C followed by 120 min at 37°C and 5 min at 85°C) using the High-capacity cDNA reverse transcription kit (Applied Biosystems). The resulting cDNA was amplified by quantitative PCR (qPCR) with an ABI Prism 7900 system (Applied Biosystems). The PCR mix was composed of 16 ng of cDNA, 250 nM of forward and reverse primers in 10 μL of 1× SYBR-Green PCR MasterMix (Applied Biosystems). Primer sequences were designed using Primer Express 3.0 software (Applied Biosystems) and salt-free purified oligonucleotides were synthesized by Integrated DNA Technologies (IDT). A full list of the primer sequences used is available in **Supplementary Table S6**.

The specificity of PCR amplification for each set of primers was checked by the presence of a single sharp peak in the melting curve analysis. Data were computed using the sequence detector software SDS 2.3 (Applied Biosystems) and analyzed using the delta-Ct relative quantification (ΔΔ Ct) method (Livak and Schmittgen, 2001) with cyclophilin and β-actin as reference genes.

### RNA-Seq Data Analysis

Raw sequencing data quality was evaluated with the application FastQC^1^.

The raw RNA-seq reads were preprocessed to remove adapter sequences using trimmomatic^2^ with the following parameters: ILLUMINACLIP: ../TruSeq3-PE-2.fa:2:151:10, LEADING:3, TRAILING:3, SLIDINGWINDOW:4:15, MINLEN:36.

For each sample, reads that passed filtering were mapped to the UCSC mouse reference genome [build mm10] using TopHat^3^ with the following changes to the default parameters described in the protocol of Trapnell et al. (2012): -G genes.gtf –no-novel-juncs –max-multihits 1. These options specified mapping to the reference genome without novel splice discovery and selection of uniquely mapped reads.

The resulting aligned reads were then analyzed with the Cufflinks suite^4^ that assembles aligned reads into transcripts and measures their relative abundance. The expression of each protein-coding gene was quantified by adding up the expression levels of all transcripts for that particular gene. Abundance is reported as the number of reads mapping to that gene divided by the gene length in kilobases and the total number of mapped reads in millions, which is called fragments per kilobase of exon per million fragments mapped (fpkm).

For further analysis, only protein-coding genes differentially expressed with a fpkm value >1 in at least one of the two conditions compared were included. The aim was to remove genes with very low expression levels, in order to minimize “background noise” and focus on more robust gene expression.

The number of independent cultures (*n*) per treatment condition used for the differential gene expression analysis differed as follows for the 1 h time point: L-lactate (*n* = 3), MK-801 (*n* = 2), MK-801 and L-lactate (*n* = 2), pyruvate (*n* = 2), NADH (*n* = 1), NMDA (*n* = 1), NMDA and L-lactate (*n* = 1); and was *n* = 2 for the 6 h time point and treatment with L-lactate.

Where more than one independent culture was used for the analysis in response to a given treatment, log_2_FC values for genes in common among the independent experiments were averaged.

### GO and Pathway Enrichment Analysis

Gene ontology (GO) and pathway enrichment analysis were performed using the clusterProfiler package (Yu et al., 2012) in the R programming environment. The functions enrichGO and enrichKEGG were utilized with Benjamini-Hochberg (BH) *p*-value adjustment (cutoff < 0.1) and the background set of genes comprised all genes with a fpkm value ≥1 in one of the two treatment conditions compared. The resulting GO terms were analyzed for semantic similarity (cut-off value of 0.7) with GOSemSim R package (Yu et al., 2010) to reduce redundancy. Selections of non-redundant enriched terms from the GO analysis were plotted. All *p*-values for plotted terms are corrected for multiple testing.

Visualization of results was performed using the dotplot function of the clusterProfiler package, and the Bioconductor packages pathview and GOplot.

### Gene Set Enrichment Analysis (GSEA)

Gene set enrichment analysis (GSEA) was conducted using WebGestalt 2017 online platform (Wang et al., 2017) run on pre-ranked list of all the genes expressed in cortical neurons with fpkm expression values >1, using the default parameters and identification of enriched categories based on false discovery rate (FDR) threshold of 0.05. If no terms were enriched below this threshold, additional terms were identified below a less stringent threshold of 0.1. Ranking was based on fold-change induction in L-lactate-treated cells compared to control cells. GSEA results were assessed as being statistically significant by permutation of 1,000 samples.

The enrichment was indicated by the net enrichment score (NES), which accounts for differences in gene set size and in correlations between gene sets and the expression dataset (Wang et al., 2017).

The leading core genes identified are genes that show high correlation between their expression level and the phenotype to which they are associated and tend to be at the extremes of the distribution, rather than randomly distributed. This subset is essentially the genes responsible for the enrichment score for a given biological process or cellular component.

### Transcription Factor Binding Site Enrichment Analysis

Detection of over-represented conserved transcription factor binding sites (TFBS) was performed with the Single Site Analysis tool on the oPOSSUM-3 website (Kwon et al., 2012) using a conservation cutoff of 0.60 and a matrix score threshold of 85%. The amount of upstream/downstream sequence analyzed was 3,000/1,000 bp. A list of all expressed genes with fpkm values >1 in cortical neurons was used for background. oPOSSUM analyzes TFBS enrichment through both *Z*-score and *F*-score, which is the negative log of the Fisher one-tailed exact probability. The *Z*-score used compares the rate of occurrence of a TFBS in the target set of genes to the expected rate estimated from the pre-computed background. The *F*-score is based on the one-tailed Fisher exact probability, which compares the proportion of co-expressed genes containing a particular TFBS to the proportion contained in the background set. Thus the *F*-score/*p*-value does not consider the number of times a TFBS appears near a gene beyond once. For enrichment of TFBS, we employed a combined threshold of 10 for *Z*-scores and 7 for *F*-scores, based on available recommendations and literature (Ahmed et al., 2017; Taylor et al., 2017).

### Cell Type-Enrichment Analysis

To estimate cell-type sources, including cell culture contaminants such as astrocytes, endothelial cells, oligodendrocytes and microglia, from which genes may originate, we used a recently published cell-type deconvolution analysis (Saul et al., 2017) using, as reference sets, RNA-seq data of sorted cells from the mouse frontal cortex (Zhang et al., 2014). Genes enriched for each cell type were selected on the basis of expression values greater than 1 fpkm and on expression in a given cell type compared to all others greater than fivefold. Overlap with the lists of differentially expressed genes in our study was evaluated.

## Results

### L-Lactate Modulates Expression of Immediate-Early Genes Involved in Transcription Regulation and Early Signaling Events

Lactate induces plasticity-related genes in cortical neurons (Yang et al., 2014). In order to gain an exhaustive insight into the transcriptional response to lactate in neurons, we have performed a genome-wide RNA-seq study.

RNA sequencing was performed on RNA isolated from DIV 11 cultured cortical neurons 1 h after addition of sodium L-lactate to cell culture media.

All treated samples used in this study had RIN values >8 (range 8.2–10) confirming that all RNA samples were of excellent quality (Ibberson et al., 2009). In total, we obtained an average of 44 million reads per sample treated with lactate for 1 h (*n* = 3 independent cultures, three biological replicates per culture). Low quality bases and adapters were filtered out from the datasets prior to read mapping. An average of 42 million of reads per sample were mapped to the mouse genome (UCSC, mm10).

Overall, around 12,000 genes were detected in neurons with expression levels above the threshold fpkm value of 1, used to separate genes with very low expression levels; this total number of expressed genes is representative for brain tissue which is characterized by the expression of more genes with less dominance of few highly expressed genes (Ramskold et al., 2009).

Differential gene expression analysis revealed that 113 genes were differentially regulated in cortical neurons treated with lactate for 1 h when compared with the control (no treatment), of which 80 genes were up-regulated and 33 were down-regulated (**Supplementary Table S1**). These genes were identified in each independent culture using a *q*-value < 0.05. The *q*-value represents the Benjamini-Hochberg multiple testing corrected *p*-value and is a measure of the FDR. Fold change values for the 113 genes are the average of the three independent cultures.

To account for possible contribution of non-neuronal cell types to gene expression changes mediated by L-lactate in our cultures, we have used a cell-type specific classification method of genes (Saul et al., 2017) based on RNA-seq data sets obtained from sorted cells of the mouse frontal cortex (Zhang et al., 2014) and examined the overlap with our list of differentially expressed genes.

Out of the 113 differentially expressed genes in response to 1 h L-lactate treatment, no astrocyte- or oligodendrocyte-enriched genes were identified in the overlaps. Two (Apold1, Kcnj2) and 6 (Calhm2, Egr2, Egr3, Gadd45b, Gm13889, and Txnip) genes were found to be, respectively, enriched in endothelial and microglial cells. Interestingly, six of these genes were also robustly expressed in neurons, while only two genes, Calhm2 and Kcnj2, had expression values below 1 fpkm in neurons (Zhang et al., 2014).

Some of the genes previously reported as modulated by lactate in neurons after a treatment of 1 h (*Arc, Egr1/Zif268, c-Fos*) were among the 113 genes identified.

Out of the 113 genes differentially regulated by lactate, 22 genes showed an average fold change above 2, indicating a strong modulation.

We next performed a GO enrichment analysis to identify over-represented biological processes, molecular functions and cellular components corresponding to the list of differentially expressed genes.

For this analysis, all differentially expressed genes with a *q*-value < 0.1 were used (135 genes – **Supplementary Table S1**). This *q*-value threshold is more lenient and can be used when aiming to maximize the discovery of relevant biological processes.

**FIGURE 1:**
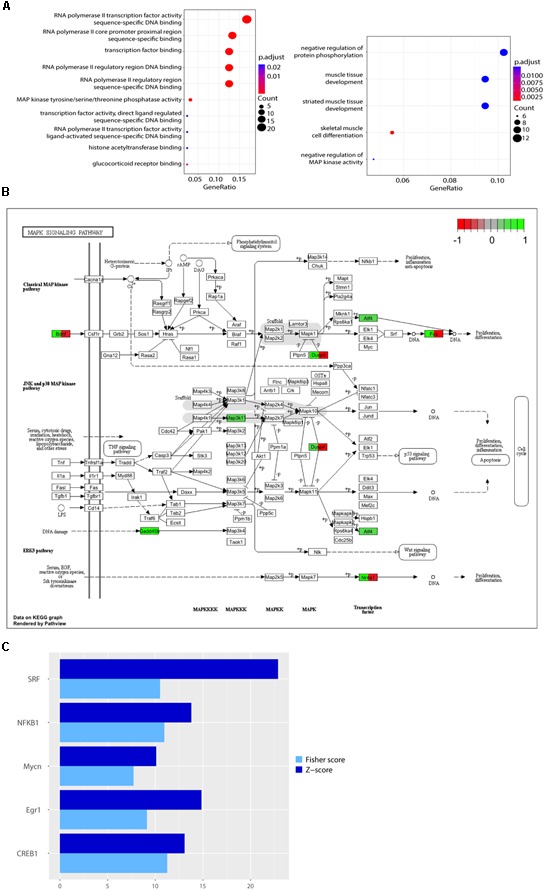
Functional analysis of genes differentially expressed in response to L-lactate after 1 h. **(A)** GO plots showing enrichment of biological processes and molecular functions for the genes differentially expressed after 1 h stimulation with L-lactate (135 genes, *q*-value < 0.1, three biological replicates, *n* = 3 independent cultures). Statistical significance for the enriched terms is based on the multiple testing-corrected *p*-value, which is color-coded. The number of genes associated with each respective term is indicated by the dot size. **(B)** A detailed KEGG graph of the MAPK signaling pathway with genes modulated by L-lactate at 1 h highlighted in green indicating that they are up-regulated. *Dusp10* and *Gadd45g* are not indicated on the pathway map. Genes highlighted in red are genes down-regulated in the co-treatment condition with MK801 and L-lactate. **(C)** Bar plot showing fold enrichment of specific transcription factor binding sites in the promoters of genes modulated by L-lactate at 1 h (FDR < 0.001). *Z*-score is the main enrichment indicator and is obtained by dividing the rate of occurrence of a TFBS in the target set of genes to the expected rate estimated from the pre-computed background. The Fisher score is calculated as the negative natural logarithm of the one-tailed Fisher exact probability.

Around 35 of the genes modulated by L-lactate at 1 h are immediate-early genes, with transcription factor activity, or genes coding for proteins involved in the regulation of phosphorylation and early signaling events, as emphasized upon conducting a GO over-representation test (**Figure 1A**). Interestingly, five out of ten of the most enriched molecular function terms point to regulation of RNA polymerase II activity. Among the most enriched GO terms representing biological processes, muscle tissue development and differentiation are included. A full list of enriched GO terms is described in **Supplementary Table S2**.

Ten out of the 135 genes differentially regulated by lactate, namely *c-Fos, Bdnf, Atf4, Nr4a1, Gadd45b, Gadd45g, Map3k11, Dusp4, Dusp6, Dusp10* are associated with the MAPK signaling pathway, a key-signaling cascade mediating, in neurons, cytoplasmic and nuclear responses to synaptic activity, and NMDAR-dependent neuronal plasticity (Sweatt, 2001; Yang et al., 2014; Bluthgen et al., 2017). They are all up-regulated by L-lactate (highlighted in green; **Figure 1B**).

### Genes Modulated by L-Lactate Are Under the Regulatory Influence of Transcription Factors That Are Induced by Synaptic Activity and NMDAR Signaling

In the context of gene expression changes by 1 h lactate treatment, we have searched for transcription factors with over-represented DNA binding site motifs in promoters of differentially expressed genes with a *q*-value < 0.1, using the oPOSSUM web application and the JASPAR database of transcription factor matrices.

The transcription factors with the highest over-representation (*Z*-score > 10 and Fisher score > 7), including SRF, Egr1, CREB1, NF-κB, and Mycn, are known to be modulated by neuronal activity and to be involved in plasticity. The SRF shows the highest enrichment in promoters of genes modulated by L-lactate at 1 h (**Figure 1C**). Interestingly, this transcription factor was previously reported in literature to play important roles in synaptic plasticity, memory and survival (Knoll and Nordheim, 2009; Stern et al., 2012). Both SRF and the cAMP response element-binding protein (CREB1), another transcription factor that is enriched in the set of genes regulated by lactate, respond to synaptic activity triggered by activation of NMDARs and the subsequent intracellular calcium increase (Sala et al., 2000; Knoll and Nordheim, 2009). Egr1, one of the most strongly up-regulated genes by lactate at 1 h, is induced in association with long-term potentiation (LTP) and after specific learning experiences, and is implicated in regulating plasticity and survival (Cole et al., 1989; Hall et al., 2001; Jones et al., 2001). NF-κB activation was also shown to be important for induction of synaptic plasticity and memory formation (Gutierrez and Davies, 2011; Engelmann and Haenold, 2016). The p50 subunit (encoded by Nfkb1, the transcription factor motif enriched in our analysis) is a subunit of the classical NF-κB pathway and is implicated in regulating axonogenesis (Haenold et al., 2014). In addition, another enriched transcription factor, Mycn, was previously reported as being induced by neuronal activity (Jopling and Willis, 2001).

A full list of enriched transcription factor motif binding sites is provided in **Supplementary Table S3**.

### The Effect of L-Lactate on Immediate-Early Gene Expression Regulation Is NMDAR Dependent

It was previously reported that L-lactate can potentiate the responses of activated NMDARs (Yang et al., 2014). To determine whether the effects of L-lactate on gene expression were mediated by NMDARs, we stimulated neurons for 1 h with lactate in the presence of the NMDARs non-competitive antagonist MK-801 (Wong et al., 1986; Huettner and Bean, 1988) or with the blocker alone, and compared the gene expression profiles. This selective NMDAR blocker was used at a concentration of 40 μM and was applied 30 min before the addition of lactate. Under these conditions NMDARs are fully blocked for the duration of the subsequent treatment (McKay et al., 2013).

MK-801 treatment alone modulated 165 genes (*q*-value < 0.05), of which 90 were up-regulated and 75 down-regulated. Among the down-regulated genes, we identified *Arc, Egr1*, and *c-Fos*, as well as *Rgs4, Rgs2*, and *Dusp6*, previously reported to be induced by co-treatment with bicuculline (BiC) and 4-aminopyridine (4-AP) and regulated in the opposite direction by MK-801 (Papadia et al., 2008). 4-AP is a K^+^ channel antagonist that enhances burst frequency (Papadia et al., 2008), and BiC is a GABA_A_ receptor antagonist that indirectly activates synaptic NMDAR by blocking synaptic inhibition (Avoli et al., 1997). These findings demonstrate that MK-801 treatment in the cortical neurons cultures down-regulates several activity-dependent genes.

A total of 275 genes were differentially regulated in cortical neurons treated with lactate and MK-801 (*q*-value < 0.05), of which 160 genes were up-regulated and 115 were down-regulated. Five genes were up-regulated with fold changes above 2 – *Txnip, Gadd45b, Bbc3, Ier5l*, and *Lpin1*. *Txnip, Bbc3*, and *Ier5* were previously identified as up-regulated in cortical neurons treated for 2 h with APV, a selective NMDAR antagonist (Chen and Hall, 2017).

*Arc, Egr1*, and *c-Fos*, genes up-regulated by lactate treatment alone, were down-regulated by the MK-801/lactate co-treatment, with fold changes below -1.5.

**FIGURE 2:**
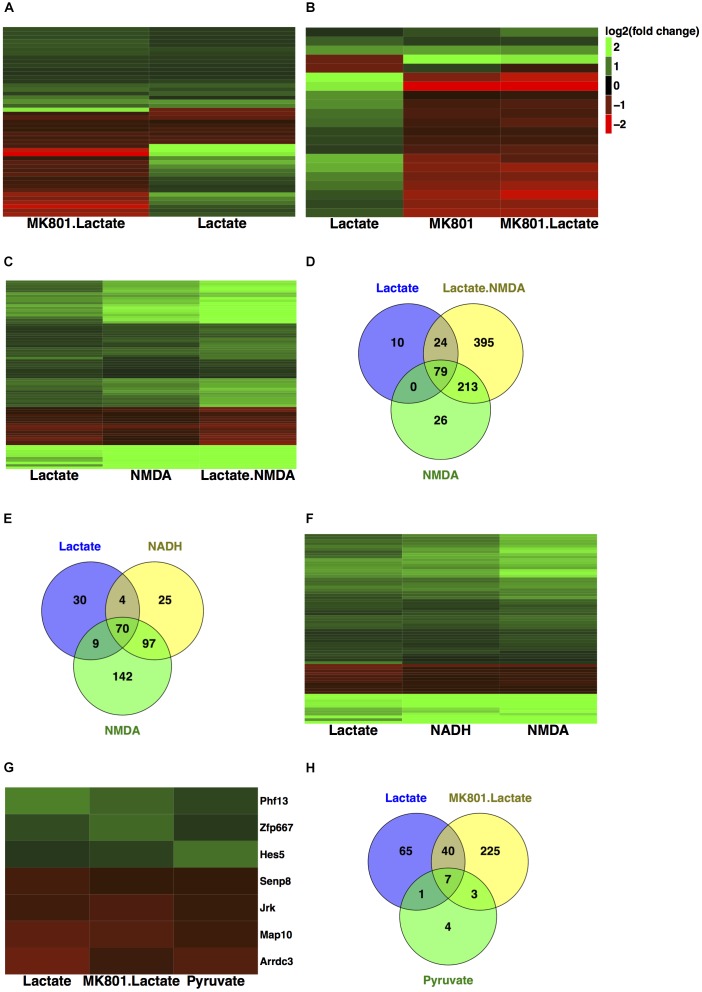
Comparison of transcriptional responses to L-lactate, MK-801, NADH, NMDA, and pyruvate. **(A)** Heatmap with genes regulated by L-lactate in absence and presence of MK-801 channel blocker in cortical neurons. Colors red and green indicate down-regulated and, respectively, up-regulated genes with the corresponding log_2_(fold change) values. **(B)** Heatmap with genes regulated by L-lactate, MK-801/L-lactate co-treatment and MK-801 alone. **(C)** Heatmap with genes modulated by L-lactate, NMDA, and L-lactate/NMDA co-treatment, after 1 h. All genes represented in the heatmap are differentially expressed in each of the treatment conditions (*q* < 0.05). **(D)** Total numbers of genes differentially expressed in response to L-lactate, NMDA, and L-lactate/NMDA co-treatment. **(E)** Total numbers of genes differentially expressed in response to L-lactate, NADH, and NMDA. **(F)** Comparison of transcriptional responses to L-lactate, NADH, and NMDA after 1 h. **(G)** Heatmap depicting genes modulated by L-lactate alone, L-lactate/MK-801 co-treatment, and pyruvate, after 1 h. **(H)** Venn diagram depicting total number of genes modulated by L-lactate, MK-801/L-lactate co-treatment and pyruvate.

When comparing expression profiles modulated by lactate and lactate together with MK-801, we found that 47 out of 113 (more than 41%) of all the genes modulated by L-lactate at 1 h were also differentially expressed when NMDARs were blocked (**Figure 2A**). Interestingly, 21 out of the 47 genes, including those with the most pronounced fold changes, showed an opposite directionality of response in the co-treatment condition with MK-801 and the condition with the blocker alone (**Figure 2B**).

**FIGURE 3:**
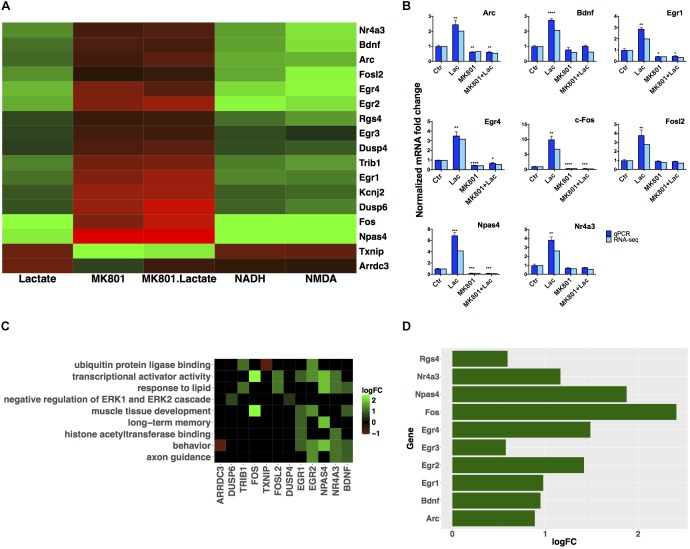
Subset of genes modulated by L-lactate. **(A)** Fold change modulation of genes regulated by L-lactate in presence and absence of MK-801, and by NADH, NMDA, and MK-801 alone. **(B)** Validation of RNA-seq data with qRT-PCR, comparison of fold expression changes between RNA-seq and qRT-PCR data for genes is shown. Error bars correspond to standard error of the mean (SEM) values. *T*-test conducted for qRT-PCR analysis, between the different treatments and Ctr condition; significance (*p* < 0.05^∗^, *p* < 0.01^∗∗^, *p* < 0.001^∗∗∗^, *p* < 0.0001^∗∗∗∗^). **(C)** Genes-terms relationship heatmap emphasizing genes associated with multiple gene ontology terms of interest and showing their fold changes expression in response to L-lactate treatment at 1 h. Up-regulated genes are indicated in green and down-regulated genes in red. All terms displayed are over-represented with a false discovery rate (FDR) < 0.05. **(D)** Bar plot showing fold induction of a few selected known synaptic plasticity-related genes. The *x*-axis shows gain of expression log_2_(fold change) 1 h after L-lactate stimulation.

Gene expression validation by quantitative real time-polymerase chain reaction (qRT-PCR) was performed for eight of these genes, and it showed good correlation with RNA-seq data (**Figure 3B**).

Five of the previously identified genes involved in the MAPK signaling pathway (*Bdnf, c-Fos, Dusp4, Dusp6, Nr4a1*) that were up-regulated by lactate treatment alone were down-regulated following co-treatment with MK-801 and L-lactate (**Figure 1B**). These observations suggest that lactate modulates selective MAPK pathway genes in a NMDAR-dependent manner.

### Transcriptional Responses to L-Lactate Are Reproduced by NADH and NMDA

Next, we aimed to validate the effects of L-lactate on gene expression mediated through the NMDAR by testing the effects of the NMDAR agonist NMDA and comparing it to that of L-lactate. Transcriptional responses 1 h after treatment with selective receptor agonist NMDA alone (50 μM) or L-lactate and NMDA co-treatment were assessed. NMDA is an established agonist of the NMDAR, which was previously shown to induce neuronal activity and modulate gene expression in cortical neurons (Thakurela et al., 2015).

We found that 79 out of the 113 genes (70%) modulated by L-lactate are similarly differentially expressed in response to NMDA alone (*q* < 0.05; **Figure 2D**). Remarkably, the genes in common between the two conditions have the same directionality of response (**Figure 2C**).

More than 91% of the genes modulated by L-lactate alone are also modulated by L-lactate and NMDA co-treatment (103 out of 113 genes; **Figure 2D**). All the fold changes for the genes in common between the two conditions are more pronounced in the case of L-lactate and NMDA co-treatment indicating additive effects (**Figure 2C**).

These observations further confirm that lactate modulates gene expression in a NMDAR-dependent manner.

Yang et al. (2014) have shown that NADH, a by-product of the metabolic processing of L-lactate to pyruvate, mimics the effect of L-lactate on the expression of *Arc, Zif268, Bdnf* and *c-Fos* genes, and that its effect is antagonized by MK-801 and follows NMDAR activation (Yang et al., 2014).

In line with these observations, we hypothesized that NADH could regulate a large proportion of the genes we see modulated by L-lactate at 1 h. Indeed, we found that approximately 65% of the total number of genes regulated by L-lactate is also modulated by NADH (74 out of 113 genes) (**Figure 2E**).

As many as 95% of the genes modulated by both L-lactate and NADH at 1 h (70 out of 74 genes), were also differentially expressed in response to NMDA treatment, an established agonist of the NMDAR (**Figure 2E**).

A very close resemblance of gene expression changes, by directionality and fold change values, was observed between the NMDA and NADH treatments (**Figure 2F**).

This suggests that for a significant proportion of genes (70 out of 113 genes), a mechanism through which L-lactate could affect gene expression is by generating an increase in NADH, which could in turn have a positive modulatory affect on NMDAR signaling.

A subgroup of genes that showed same directionality of response to L-lactate, NADH and NMDA, and an opposite directionality upon co-treatment with L-lactate and MK-801 or the blocker alone, was identified (**Figure 3A**). These 17 genes have been differentially expressed in each of the treatment conditions described above with a *q*-value < 0.05.

For these genes, we suggest that they are modulated by L-lactate through downstream NADH increase, which in turn potentiates NMDAR activity via a possible redox-dependent mechanism (Jourdain et al., 2018). The effect of L-lactate on these genes, with one exception only, is MK-801-sensitive and NMDAR-dependent.

The exception is *Arrdc3*, a gene coding for the arrestin-domain containing protein 3, which is modulated more strongly by L-lactate compared to both NADH and NMDA, and for which the effect of L-lactate on its expression is diminished but still persists upon NMDAR blockade (**Figure 3A**).

We further conducted a GO over-representation analysis for this subgroup of 17 genes. We found that several genes are transcriptional activators, of which *Egr1, Egr2, Npas4*, and *Nr4a3* are implicated in processes such as memory and behavior, and that also, some of these genes are associated with muscle tissue development: *Egr1, Egr2, c-Fos* (**Figure 3C**).

Next, in order to gain further insights into the biological functions of the NMDAR- and NADH-dependent genes, and based on the previous observations that L-lactate stimulates synaptic plasticity genes (Yang et al., 2014), we have performed a literature search to identify other genes from this gene set which could be involved in promoting synaptic plasticity. We were able to identify six additional genes (Egr2, Egr3, Egr4, Npas4, Nr4a3, Rgs4), which participate in synaptic plasticity-related processes (Atluri et al., 2013; Gerber et al., 2016). A list with corresponding fold changes is included in **Figure 3D**.

### Pyruvate Reproduces Only a Few of the Modulatory Effects of L-Lactate on Immediate-Early Genes

When comparing transcriptional responses to L-lactate alone and to L-lactate together with MK-801, we observed that about half of the genes differentially expressed in both conditions showed the same directionality of response (**Figure 2A**). This provides an indication that there could be non-NMDAR-dependent mechanisms responsible for the effects of L-lactate on gene expression.

As L-lactate is converted into pyruvate by L-lactate dehydrogenase, some of these effects could be mediated by pyruvate level increases. It is well known that astrocyte-derived lactate is taken up by neurons to fuel their energy needs, a process in which lactate is converted to pyruvate, which is then oxidized to CO_2_ and water in the TCA cycle to produce ATP (Laughton et al., 2007; Wyss et al., 2011; Brooks and Martin, 2015).

To determine whether some of the effects of lactate on gene expression could be due to changes in energy metabolism, we tested whether pyruvate could mimic effects of L-lactate on gene expression.

In cortical neurons, 1 h of pyruvate treatment modulated the expression of 15 genes, of which 11 were down-regulated and 4 genes up-regulated (*q*-value < 0.05).

Among the genes regulated by pyruvate, we found that eight genes were also significantly modulated by L-lactate (**Figure 2H**). Interestingly, however, as shown in **Figure 2G**, the expression of seven of these genes (*Phf13, Zfp667, Hes5, Senp8, Jrk, Map10, Arrdc3*) was not affected by the NMDAR blocker when co-applied with lactate. This comparison suggests that the effect of L-lactate on the expression of these genes could be due to an increase in the oxidative energy metabolism via pyruvate and its subsequent use by the TCA cycle, but not through activation of NMDARs.

### Gene Set Enrichment Analysis Reveals Epigenetic Mediators That Are Regulated by L-Lactate and Gene Targets Linked to Various Phenotypes

In order to achieve additional insights into biological mechanisms modulated by L-lactate, gene set enrichment analysis (GSEA) was performed on the full list of genes robustly expressed (fpkm > 1) in cortical neurons treated with L-lactate after 1 h (12,259 genes). This analysis takes into account genes with low or non-differentially expressed values that can participate in final enrichment through cumulative effects (Subramanian et al., 2005; Fruzangohar et al., 2017).

The GSEA conducted on the 12,259 genes ranked by fold change and using the GO functional database, revealed a positive enrichment of histone acetyltransferase binding genes and a negative enrichment of DNA packaging complex and protein-DNA complex genes. The leading core genes, genes which contributed the most to the enrichment signal calculation for a given GO term (described in “Materials and Methods” section), are listed in **Table 1**. A full list of ontology terms enriched is included in **Supplementary Table S4**.

**Table 1:** Enriched gene ontology terms as assessed by gene set enrichment analysis of genes expressed after 1 h of L-lactate treatment.

Gene ontology term	Net enrichment score (NES)	False discovery Rate (FDR)	Leading core genes
Histone acetyltransferase binding	1.92	0.04	Cebpb; Egrl; Zbtb7a; Cited2; Nr4a3
DNA packaging complex	-2.38	0.00	Histlh4m; Hlf0; Hist2h3cl; Hist2h2aal; Histlh2bk; Histlh2bm; Histlh2bp; Hist2h2bb; Hist2h2be; Histlhlc
Protein–DNA complex	-1.99	0.01	Histlh4m; Gins4; Hlf0; Hist2h3cl; Hist2h2aal; Nfe212; Nfya; Nfyb; Poldl; Pole2; Gtf2h3; Terfl; Xpa; Gins2; Hist3h2a; Histlh2bk; Histlh2bm; Histlh2bp; Hist2h2bb; Hist2h2be; Histlhlc; Rpa3

*A positive net enrichment score indicates that genes over-represented associated with the respective term are up-regulated in the expression dataset. Negative NES indicates the opposite*.

It was previously reported that L-lactate is a weak histone deacetylase inhibitor (Latham et al., 2012) and that it can induce histone H3 and H4 hyperacetylation and decrease chromatin compactness in HeLa cells (Wagner et al., 2015).

The same type of analysis conducted on the 12,259 genes using the mammalian phenotype ontology database, revealed enrichment of a series of phenotypes which have been previously linked to the Astrocyte Neuron Lactate Shuttle (ANLS) (Magistretti and Allaman, 2018) and lactate, such as learning (Tadi et al., 2015; Shannon et al., 2016; Jin et al., 2017) and sleep (Naylor et al., 2012; Petit et al., 2013; Haydon, 2017), and others for which there are indications of lactate involvement such as hyperactivity and anxiety disorders (Dillon et al., 1987; Russell et al., 2006; Johnson et al., 2008; Killeen et al., 2013).

The leading core genes accounting for the phenotypes enrichment signal are listed in **Table 2**. A full list of terms enriched is included in **Supplementary Table S4**.

**Table 2:** Gene targets of L-lactate linked to selected phenotypes by gene set enrichment analysis.

Phenotype	NES	FDR	Leading core genes
Abnormal NMDA-mediated synaptic currents	2.10	0.03	Fosb
Impaired spatial learning	2.13	0.03	Fosb
Abnormal paradoxical sleep pattern	2.08	0.03	Fos; Fosb
Abnormal sleep pattern	2.28	0.00	Fos; Fosb; Perl; Per2; Ptchdl; Slcl8a2
Impaired learning	2.29	0.00	Arx; Fmrl; Fosb; Nr4a2; Syt4; Ptchdl; Tnc
Abnormal sleep behavior	2.14	0.03	Fos; Fosb; Perl; Per2; Ptchdl; Slcl8a2; Foxo3
Abnormal interleukin-4 secretion	2.09	0.03	Atf3; Cish; Gadd45b; Nfatcl; Nfil3; Dusp4; Rnfl28
Decreased dopamine level	2.11	0.03	Gpr37; Hipk2; Nr4a2; Perl; Ptgerl; Sstr2; Slcl8a2; Tnc
Abnormal tumor necrosis factor level	2.21	0.01	Ticam; Cebpb; Crebbp; Egrl; Fos; Ier3; Ldlr; Mapkapk2; Mertk; Ncoa3; Nfil3; Npylr; Pcskl; Duspl; Relb; Arid5a; Tnfrsfla; Pde4d; Sh3bp2; Rc3hl; Tbkl; Apba3; Pelil
Abnormal fear/anxiety-related behavior	2.15	0.03	Spred; Adcyap1; Arc; Arx; Bdnf; Crem; Arid3a; Fmr1; Fos; Fosb; Gpr19; Grin2a; Nr4a2; Penk; Dusp1; Rgs2; Slc2a3; Sstr2; Cpeb3; Slc18a2; Git1; Tnc; Npas4; Gpr26; Dcaf10; B3galt2; Park2; Lrrtm1
Hyperactivity	2.13	0.03	Braf; Adcyapl; Arx; Bdnf; Chrm4; Crem; Fmrl; Fos; Fosb; Ldlr; Nr4a3; Npylr; Nr4a2; Perl; Lrrc4; Rgs4; Slcl2a2; Ptchdl; Gitl; Tnc; Npas4; Sik2; B3galt2; Dnajb9; Vgf; Usp2; Fezf2; Cystml; Adipor2; Ssfa2; Gpr22

### On the Long-Term L-Lactate Modulates Genes Involved in Receptor Signaling and Neuronal Excitability

In order to investigate how the transcriptome of cortical neurons is regulated by L-lactate on the long-term, differentially expressed genes in cortical neurons after 6 h treatment with L-lactate were identified.

In total, 313 genes were differentially expressed, of which 112 were down-regulated and 201 were up-regulated (Benjamini-Hochberg adjusted *p*-value < 0.05). Fold change values for the majority of these genes were below 1.5, which indicates weak modulation.

Among these 313 genes, 8 are enriched in astrocytes (Dio2, Fras1, Gfap, Mtmr11, Ngef, Notch3, Ppp1r3c, Thbs1), 9 in endothelial cells (Apln, Cd55, Lrrc55, Net1, Odc1, Slc39a10, Tes, Tfrc, Usp43), 3 in microglia (Rtn4rl1, St6galnac4, Txnip), and 3 in oligodendrocytes (Bcas1, Dnph1, Plp1).

Some of the up-regulated genes with the strongest modulation were *Vegfa* (vascular endothelial growth factor-coding gene), *Synj2* (synaptojanin 2) and *Baiap2l2* (brain specific angiogenesis inhibitor 1-associated protein 2-like protein 2).

We next performed a GO over-representation test on all differentially expressed genes with a *q*-value < 0.1 (in total 484 genes – **Supplementary Table S5**).

**FIGURE 4:**
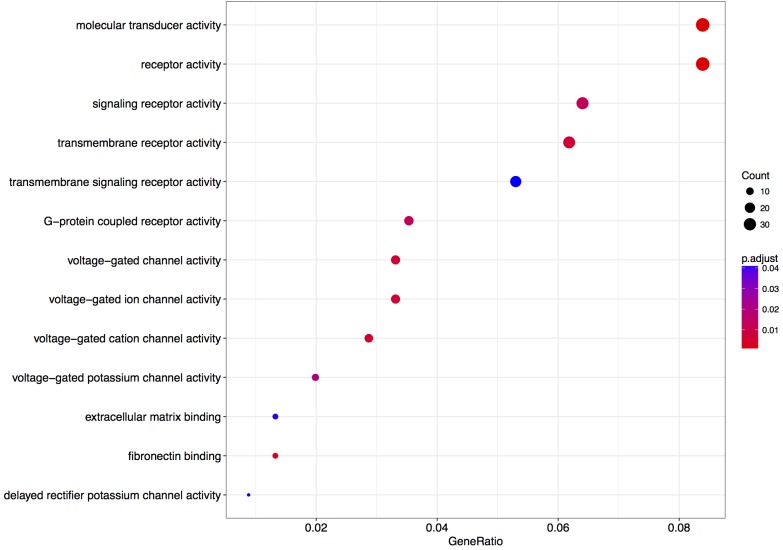
Enriched gene ontology terms 6 h after L-lactate stimulation. GO plot shows enrichment of biological processes and molecular functions for genes modulated in cortical neurons by L-lactate after 6 h.

The enrichment of genes coding for voltage-gated cation channel activity proteins and potassium channels was observed (**Figure 4**). Few of the terms are associated with receptor-mediated signaling events, which could be associated with synaptic plasticity phenomena.

In addition to the GO analysis, we have performed a gene set enrichment analysis on all genes robustly expressed (fpkm > 1) in cortical neurons in the treatment condition with L-lactate after 6 h (12,462 genes).

A positive enrichment of genes located at the synaptic membrane and dendritic processes was observed, along with a negative enrichment of genes coding for members of the cytochrome complex, the mitochondrial matrix, and the DNA packaging complex (**Table 3**).

**Table 3:** Enriched gene ontology terms as assessed by gene set enrichment analysis of genes expressed after 6 h of L-lactate treatment.

Gene ontology ID	Description	NES	FDR	Core genes
GO:0030425	Dendrite	1.62	0.07	**Supplementary Table S4**
GO:0097060	Synaptic membrane	1.62	0.07	**Supplementary Table S4**
GO:0070069	Cytochrome complex	-1.73	0.08	Uqcc3; Cox6a2; Cox7c; Cox8b; Ndufa4; Coxl5; Uqcrl0; Coxl0
GO:0005759	Mitochondrial matrix	-1.79	0.08	**Supplementary Table S4**
GO:0044815	DNA packaging complex	-1.79	0.08	Cenpa; Smc2; Hist2h2aal; H2afj; Hlfx; Hist3h2a; Histlh2bk; Histlh2bm; Histlh2bp; Hist2h2bb; Hist2h2be; Cenpt; H2afy2; Histlhlc; Ncapd2; Hist3h2ba; Ncapd3

## Discussion

In this study, we report a transcriptional analysis of gene expression modulation in neurons evoked by exposure to L-lactate. Taken together, the results obtained expand in an unbiased manner and refine the findings of the previous investigation made on candidate genes which demonstrated that L-lactate potentiates NMDA signaling in neurons, promotes expression of selected plasticity genes, and that NADH reproduces the effects of L-lactate on NMDA signaling (Yang et al., 2014). In addition, the current study shows that L-lactate and NADH regulate a majority of immediate early genes (70 out of 113 genes) in a similar way as the NMDAR agonist, NMDA.

This study has identified six synaptic plasticity-promoting genes – in addition to *Arc, Egr1, c-Fos* and *Bdnf* (Yang et al., 2014) – namely: *Egr2, Egr3, Egr4, Npas4, Nr4a3, Rgs4* which are modulated by L-lactate in cortical neurons, and 10 genes associated with the MAPK signaling pathway, a key-signaling cascade mediating cytoplasmic and nuclear responses to synaptic activity, and NMDAR-dependent neuronal plasticity, namely: *c-Fos, Bdnf, Atf4, Nr4a1, Gadd45b, Gadd45g, Map3k11, Dusp4, Dusp6, Dusp10* (Sweatt, 2001; Yang et al., 2014; Bluthgen et al., 2017).

In a previous study, Zhang et al. (2007) showed data indicating that the sub-cellular location of the activated NMDARs (synaptic vs. extra-synaptic) specifies the phenotype of the transcriptional response. Stimulation of synaptic NMDARs up-regulates pro-survival genes and down-regulates pro-death genes, while activation of extra-synaptic NMDARs leads to an opposite effect (Zhang et al., 2007).

Interestingly, among the 113 genes significantly modulated by L-lactate after 1 h (*q*-value < 0.1), 13 genes (*1190002N15Rik, Atf4, Dusp6, Dusp10, Fosl2, Frmd6, Junb, Maff, Nfil3, Npas4, Nr4a1, Pim3, Trib1*) were previously reported as regulated by a stimulation protocol known to specifically activate synaptic NMDARs (Zhang et al., 2007), whereas none are common with genes regulated by stimulating extra-synaptic NMDARs (Zhang et al., 2007).

In addition, five out of the eight genes previously reported (Papadia et al., 2008) as modulated by BiC and 4-AP in a MK-801 sensitive manner, both *in vitro* and *in vivo*, are significantly modulated by 1 h L-lactate treatment with the same pattern of regulation: *Dusp6, Egr1, Rgs2, Rgs4*, and *Txnip*. It was previously shown that BiC/4-AP treatment triggers activation of synaptic but not extra-synaptic NMDARs (Hardingham et al., 2002). Together, these observations suggest that lactate may act preferentially on synaptically localized NMDARs regulating transcriptome profile favoring neuroprotection and plasticity.

A transcription factor motifs binding sites analysis revealed that a majority of the genes modulated by L-lactate at 1 h are under the regulatory influence of transcription factors, which are induced by synaptic activity and mediate plasticity events (SRF, CREB1, Egr1, NF-κB, Mycn). Remarkably, the gene coding for Egr1, one of the transcription factors with over-represented binding sites, is itself up-regulated by L-lactate after 1 h (fold change of 1.97), and could well represent a master regulator of gene expression in response to L-lactate.

The set of 16 genes identified (excluding *Arrdc3*) for which there is strong evidence of a NADH-mediated and MK-801-sensitive mechanism (**Figure 3A**), appear as representing a “canonical” set of early genes modulated by L-lactate in cortical neurons in a NMDAR-dependent manner.

*Arrdc3* modulation showed an intriguing feature, presenting an opposite directionality of response with the blocker alone compared to L-lactate treatment, but the same directionality of response in the L-lactate treatment condition and the co-treatment condition with MK-801. This partial insensitivity to MK-801 in presence of L-lactate could be explained by the fact that pyruvate was also found to modulate this gene and thus the effect of L-lactate on its regulation could be a metabolic and not an NMDAR/NADH-dependent one. *Arrdc3* regulates the endosomal residence time and intracellular signaling of the β_2_ adrenergic receptor in HEK293 and BT549 cancer cell lines (Patwari et al., 2011; Tian et al., 2016), however, to our knowledge, there is no evidence in the literature for a role in the brain, where its transcript levels were found to be very low (Patwari et al., 2011).

In this study, we have identified in total seven genes whose expression is modulated by pyruvate: *Phf13, Zfp667, Hes5, Senp8, Jrk, Map10*, and *Arrdc3*.

One mechanism through which NADH reproduces L-lactate-mediated gene expression could rely on its ability to positively modulate NMDAR activity through its redox-sensitive regulatory sites (Choi et al., 2001; Jourdain et al., 2018). Consistent with this, NADH has been previously shown to trigger an MK-801-sensitive (hence NMDAR-mediated) rise in intracellular calcium and to amplify NMDAR-mediated currents (Yang et al., 2014).

A previous study reported that rapid activity-induced transcription of IEGs in neurons relies on elevated RNA polymerase II presence at promoters through stalling (Saha et al., 2011). It is notable, that some of the most enriched GO terms for genes modulated after 1 h by L-lactate are associated with RNA polymerase II activity, binding, and regulation. This evidence could point to a mechanism responsible for IEGs induction by L-lactate, upon NMDAR potentiation.

It is also of note that among the most enriched GO terms for biological processes, muscle tissue development and differentiation are included. This may reflect the presence of neurotrophin-coding genes which have also been reported to play an important role in muscle differentiation and regeneration (Clow and Jasmin, 2010), and could indicate a conserved gene regulatory role of L-lactate in muscle and the brain.

The enrichment of genes coding for voltage-gated cation channel activity proteins and potassium channels after 6 h, point to long-term signaling effects of L-lactate on regulation of neuronal excitability (Sjostrom et al., 2008; Shah and Aizenman, 2014). The positive enrichment of genes located at the synaptic membrane and dendritic processes, points to effects on synaptic plasticity processes. Among the genes with the highest fold change response to L-lactate after 6 h, *Vegfa* is known to stimulate neuronal survival and axonal outgrowth (Carmeliet and Storkebaum, 2002; Theis and Theiss, 2018), while *Synj2* is implicated in recycling neurotransmitter vesicles (Planchart, 2013), and *Baiap2l2*’s homolog *Baiap2* has been implicated in dendritic spine development and NMDAR regulation (Kang et al., 2016).

Apart from *Vegfa*, other genes implicated in neuronal survival and neuroprotection were modulated by L-lactate after 1 h, namely *Nr4a2, Npas4/Nxf, Bdnf*, and *Bcl2l11/Bim*, and after 6 h: *Adcyap1, Bdnf, Apaf1, Bim, Gfra2, Hrk* (**Table 4**). Interestingly, *Hrk, Apaf1*, and *Bim* that are key factors in promoting neuronal cell death (Cheung et al., 2005; Hetz et al., 2007; Steckley et al., 2007; Ghosh et al., 2011) were down-regulated by L-lactate, while *Bdnf, Nr4a2, Npas4, Adcyap1*, and *Gfra2*, that facilitate neuronal survival (Lahteenmaki et al., 2007) and protection (Vaudry et al., 2002; Husson et al., 2005; Shintani et al., 2005; Ooe et al., 2009; Volakakis et al., 2010) were up-regulated by L-lactate. Furthermore, L-lactate treatment for 1 h strongly down- regulated (absolute fold change of 2) *Txnip*, a gene coding for an endogenous inhibitor of the antioxidative function of thioredoxin. *Txnip* upregulation was associated with stroke and other neurological conditions and its inhibition was shown to be neuroprotective (Al-Gayyar et al., 2011; Nasoohi et al., 2018). These observations collectively suggest a role for L-lactate in modulating a neuroprotective transcriptional program.

**Table 4:** Neuroprotection and pro-apoptotic genes modulated by L-lactate after 1 h and 6 h treatment.

Function	Gene symbol	Log_2_FC	Time
Neuroprotection	Nr4a2	1.91	1 h
	Npas4	1.88	1 h
	Bdnf	0.95 0.40	1 h 6 h
	Vegfa	0.83	6 h
	Adcyapl	0.53	6 h
	Gfra2	0.30	6 h
Cell death	Txnip	-0.99	1 h
	Apafl	-0.23	6 h
	Bcl2111	-0.35	6 h
	Hrk	-0.34	6 h

A number of genes significantly modulated by L-lactate after 1 h are not mediated through NMDAR signaling, or through NADH or pyruvate increases. They could be regulated through epigenetic mechanisms linked to L-lactate’s weak histone deacetylase activity inhibitory role, previously reported in literature (Latham et al., 2012). Gene set enrichment analysis results have provided indications that L-lactate modulates genes coding for histone acetyltransferase binding proteins, and histone protein complexes, which could in turn impact chromatin compactness and organization, and gene expression.

The phenotypes identified as enriched for the list of genes modulated by L-lactate at 1 h point to a series of genes relevant to sleep physiology and psychiatric conditions such as anxiety and hyperactivity that open avenues for further investigation.

## Conclusion

**FIGURE 5:**
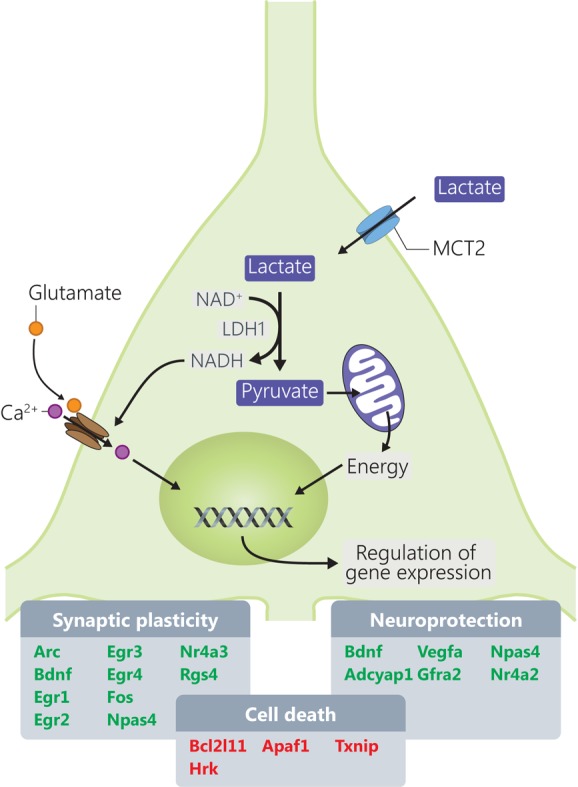
Mechanisms through which L-lactate modulates mRNA expression of genes involved in synaptic plasticity and neuroprotection. Genes highlighted in red and green are downregulated, respectively, upregulated by L-lactate.

The present study provides a molecular dissection of the transcriptome-level responses to L-lactate in cortical neurons leading to identification of several groups of genes that are responsive to this energy metabolite and signaling molecule (Magistretti and Allaman, 2018) and that are important for the physiology of neurons and processes like synaptic plasticity and neuroprotection (**Figure 5**).

## Accession Code

Fastq files were uploaded to NCBI SRA database with accession number SRP150704.

## Author Contributions

HF and PM designed the research. HF and MM performed the research. MM, HF, and HM analyzed the data. MM, HF, and PM wrote the paper.

## Conflict of Interest Statement

The authors declare that the research was conducted in the absence of any commercial or financial relationships that could be construed as a potential conflict of interest.

## Footnotes

1 http://www.bioinformatics.babraham.ac.uk/projects/fastqc/

2 http://www.usadellab.org/cms/?page=trimmomatic

3 http://ccb.jhu.edu/software/tophat/index.shtml

4 http://cufflinks.cbcb.umd.edu
